# Copy Number Variation and Selection Signal: Exploring the Domestication History and Phenotype Differences Between Duroc and the Chinese Native Ningxiang Pigs

**DOI:** 10.3390/ijms252111716

**Published:** 2024-10-31

**Authors:** Fang Yang, Wenwu Chen, Yanda Yang, Yang Meng, Yantong Chen, Xiaoling Ding, Yuebo Zhang, Jun He, Ning Gao

**Affiliations:** 1 Key Laboratory of Livestock and Poultry Resources (Pig) Evaluation and Utilization, Ministry of Agriculture and Rural Affairs, College of Animal Science and Technology, Hunan Agricultural University, Changsha 410128, China; y621829@163.com (F.Y.); cww1242646778@163.com (W.C.); ydyang@stu.hunau.edu.cn (Y.Y.); 15188234010@163.com (Y.M.); chenyt0298@163.com (Y.C.); ybzhangfd@126.com (Y.Z.); 2 Yuelushan Laboratory, Changsha 410128, China; 3 College of Animal Science and Technology, Anhui Agricultural University, Hefei 230036, China; dinxiaoling1997@ahau.edu.cn

**Keywords:** Ningxiang pigs, CNVs, selective signals, phenotypic variation, QTL

## Abstract

The Ningxiang pig, one of the well-known Chinese native pig breeds, has the advantages of tender meat, high intramuscular fat (IMF) content, and roughage tolerance, compared to the commercial lean pig breeds. The genetic basis for complex traits in Ningxiang pigs has been previously studied through other genetic markers, such as Single Nucleotide Polymorphism (SNP), while the characteristics of copy number variation (CNV) and the selection signal have not been investigated yet. In this study, GGP 50 k genotyping data of 2242 Ningxiang pigs (NX) and 1137 Duroc pigs (Duroc) were involved in CNV atlas construction and selection signals identification. Annotations of genes and quantitative trait locus (QTLs) were performed on the target candidate regions, as follows: (1) 162 CNVs were detected in Ningxiang pigs, while 326 CNVs were detected in Duroc pigs, and there are 21 copy number variation regions (CNVRs) shared between them; (2) The CNVRs of Duroc are more abundant, with 192 CNVRs, accounting for 1.61% of the entire genome, while those of Ningxiang pigs only have 98 CNVRs, accounting for 0.49%; (3) The QTLs annotated on CNVs and selected regions of Ningxiang pigs were mainly associated with meat quality and fertility. In contrast, the Duroc QTLs’ notes relate primarily to the carcass and immunity, and explain why they have a higher slaughter rate and immunity; (4) There is a presence of high-frequency acquired CNVs, specifically in Ningxiang pigs, with 24 genes significantly enriched in the sensory receptor-related pathway in this region; (5) Based on the CNVs atlas, candidate genes such as 3 inositol 1,4,5-triphosphate receptor, type 3 (*ITPR3*), forkhead box protein K2 (*FOXK2*), G-protein coupled estrogen receptor 1 (*GPER1*), Glyceraldehyde 3-phosphate dehydrogenase (*GAPDH*), triosephosphate isomerase 1 (*TPI1*), and other candidate genes related to fat deposition and differentiation were screened. In general, this study improved our knowledge about copy number variation and selection signal information of Ningxiang pigs, which can not only further explain the genetic differences between Chinese native and Western commercial pig breeds, but also provide new materials for the analysis of the genetic basis of complex traits.

## 1. Introduction

Pork is a vital source of animal protein for global human consumption, and it is China’s most widely consumed meat product. Research has shown that there is a significant inconsistency between Chinese native pigs and lean pig breeds in terms of disease resistance [1], growth, and development [2]. Ningxiang pigs (NX), a famous local fat-type pig breed in China, have high-quality intramuscular fat (IMF) content [3]. Duroc is widely chosen as a high-quality paternal breed due to its’ excellent meat quality and fast growth rate [4]. Moreover, Duroc, as an ancient domesticated pig breed, was subjected to strong directional selection because of changes in the environment and breeding requirements [5]. This is completely different from the development history of the local pig breeding population in China. However, most domestic pigs in China have disadvantages, such as slow growth, low feed utilization efficiency, and poor reproductive ability [6]. The significant differences in phenotype and production traits between the two varieties have been reported, especially in the growth rate and size of NX in relation to Duroc [7]. To address this problem, breeders have recently conducted a targeted selection of individuals within certain varieties, based on existing knowledge of their genetic background. After a certain amount of effort, the carcass traits of NX have been improved [8].

The genetic diversity of a population is influenced by several factors, including genetic variation and positive selection. Genetic variation is a direct contribution to phenotypic variation, and it encompasses a single nucleotide site and segmental variation on the genome. Copy number variations (CNVs) represent a variation in the number of duplicate copies of a DNA sequence in the genome, causing differences in the copy number (CN) among individuals or populations [9]. Furthermore, it has a longer length and can involve DNA sequences of thousands to millions of base pairs [10]. Normally, CNVs influence phenotype through molecular regulatory mechanisms such as dose utilization, regulatory sequence transfer or alteration, and gene fusion [11]. These mechanisms are a necessary condition for CNV to explain biological phenomena such as genetic diseases and population evolution. In recent years, numerous studies have been conducted on the CNVs within pig genomes, leading to the detection and identification of many CNVs associated with significant economic traits. For example, CNVs play roles in shaping reproductive traits [12], and differences in CNVs between Chinese and Western pig breeds can lead to differences in meat quality [13], growth traits [14], and coat color [15]. Therefore, obtaining the copy number variation information of the entire genome of NXs not only improves the genetic variation information, but also allows for a comparison with Duroc, resulting in a further understanding of the causal relationship between genetic background and phenotypic differences.

Positive selection occurs and indirectly contributes to phenotypic change. In genetic evolution, positive selection usually refers to the selection of genotypes under specific environmental conditions [16]. These signals may include the physical or biological environment (such as the presence of predators), as well as, possibly, the domestication of the species. With the development of current technology, it has been reported that a profound phylogenetic split occurred between Asian and European pigs 1 million years ago, and that the unique phenotypic characteristics of Asian pigs and European pigs are due to the independent domestication of the two regions [17]. Meanwhile, selection is also an important method of genetic improvement and new breed cultivation in pigs. Artificial selection will result in different unique traits between pig breeds of the two regions [18]. Commercial pig breeds, such as Duroc, were developed early on as a pristine biological resource to drive the meat industry, and they experienced significant artificial and environmental selection pressures in terms of growth rate and body size to meet production and social needs [19]. However, due to social development and breed characteristics, Chinese domestic pig breeds have only recently been widely selected for breeding. The research on the genetic basis of germplasm characteristics is not deep enough, compared to commercial pigs with a longer domestication time.

To better understand the contribution of genetic variation and selection to the differences between Duroc and NX breeds, we used highly dense SNP data to construct a CNV atlas for the two pig breeds. In order to avoid the negative impact of non-purebred NXs on the accuracy of atlas construction, the method of estimating the genomic breed composition (GBC) was adopted to retain purebred individuals as much as possible for atlas construction. Although research reports on NXs are increasing, research on their copy number variation has not emerged yet. This study fills a gap in the study of copy number variation in NXs, and also screens out regions of the genome that may be under selection. By conducting further comparisons and gene annotations between the two breeds, this study analyzed the potential impacts of structural variation and selection pressure on the phenotypes of these pig breeds. The findings provide new genetic variation data for enhancing the genetic quality of NX pigs, as well as offering valuable insights for breeders to optimize their breeding programs.

## 2. Results

### 2.1. Identification and Characterization of CNVs

**Figure 1:**
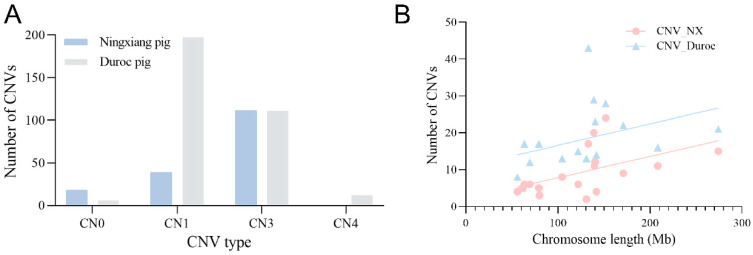
CNV characteristics of NX and Duroc pigs. The number of different CNV-type distributions (**A**); the relationship between CNV number and chromosome length (**B**).

A CNV assay and filtering were performed on the post-QC samples. Of them, we detected 168 CNVs in 834 NX, and identified a total number of 326 CNVs for 828 Duroc pigs. The R-CNVs of the two populations were 69.85% (NX) and 81.90% (Duroc), respectively (Table 1). There were differences in the distribution of CNV types and characteristics between the two groups. No CN4-type CNVs were found in the NX, and the other 3 types accounted for 10.71% (CN0), 23.21% (CN1), and 66.07% (CN3), respectively, CN3 accounted for the highest proportion. Duroc has the largest proportion of CN1 (60.43%), followed by CN3 (34.05%), CN4 (3.68%), and CN0 (1.84%) (Figure 1A, Table S1). Through regression analysis, we identified a significant positive linear correlation between the length of chromosome sequences and the CNV present on them (*p* = 0.0277) (Figure 1B). There is a significant positive correlation between the number of CNVs and the chromosome length in NX and Duroc (*p* < 0.05).

**Figure 2:**
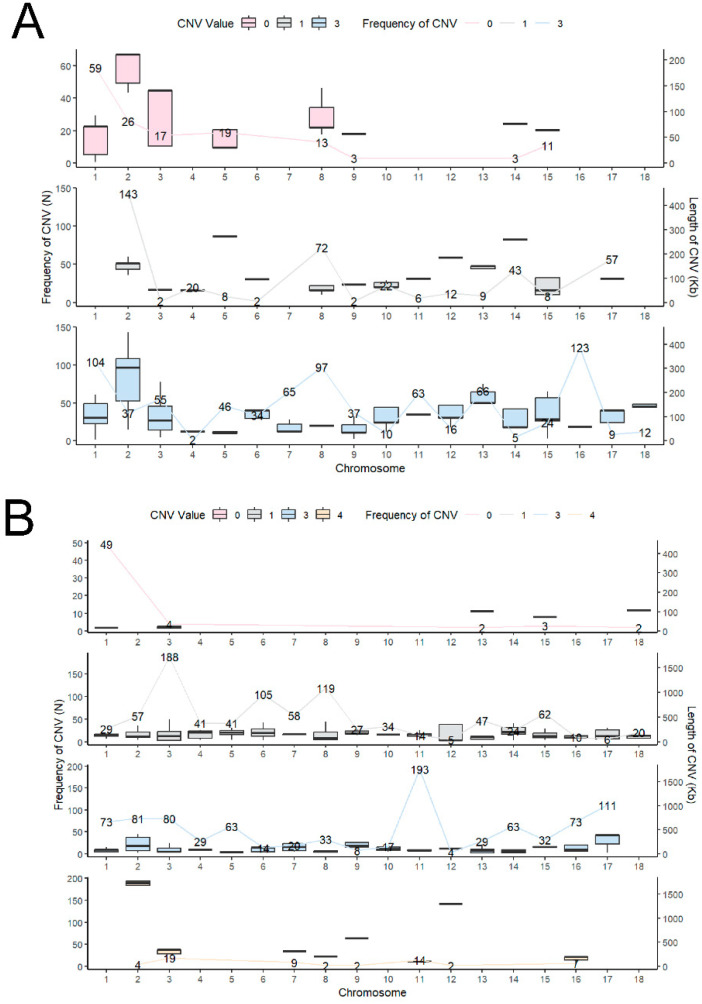
CNV characteristics of NX and Duroc. Distribution frequency of different types of CNV on chromosomes of NX and Duroc pigs (**A**,**B**).

There were significant differences in the frequency and type of copy number occurrence on different chromosomes, along with population heterogeneity (Figure 2A,B). SSC1, SSC2, SSC3, SSC8, and SSC11 were the chromosomes with a high CNV incidence. SSC1, in particular, was the main chromosome for the CN0-type variation in the two breeds. CN1 and CN3 of NX were mainly distributed between SSC2 and SSC16, respectively (Figure 2A), while CN1 and CN3 of Duroc were distributed between SSC3 and SSC11, respectively (Figure 2B). Regarding the length of the copy number variant fragments, the CNV sizes ranged from 1.65–450.313 kb for NX, and from 14.323–1763.94 kb for Duroc.

### 2.2. Comparative Analysis of CNVRs in NX and Duroc Pigs

**Figure 3:**
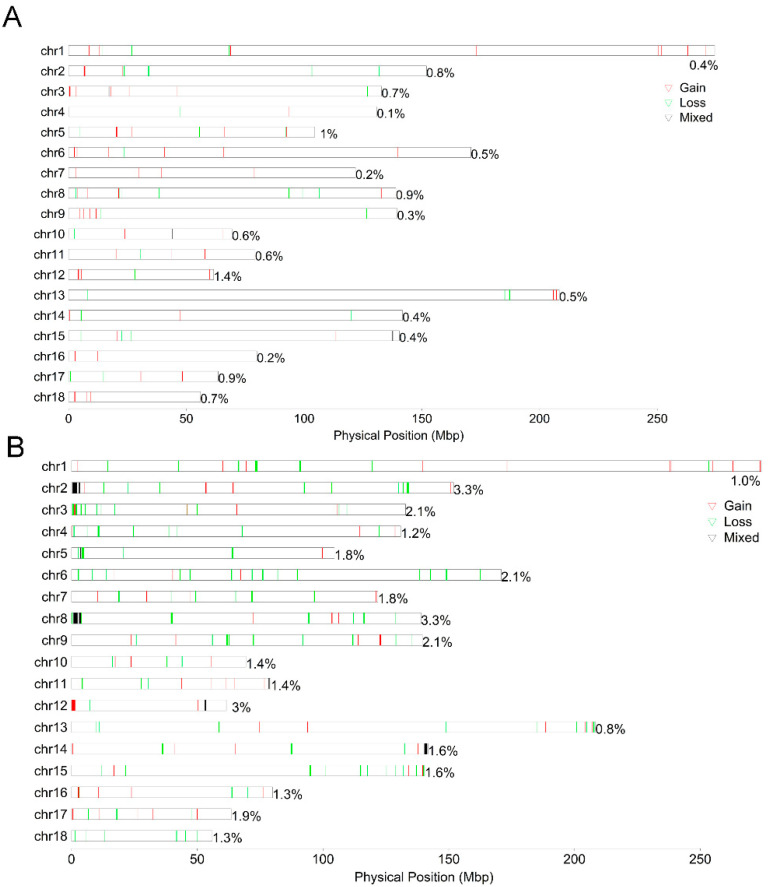
The composition and distribution of CNVRs. Density of CNVRs per chromosome in NX (**A**) and Duroc (**B**).

**Figure 4:**
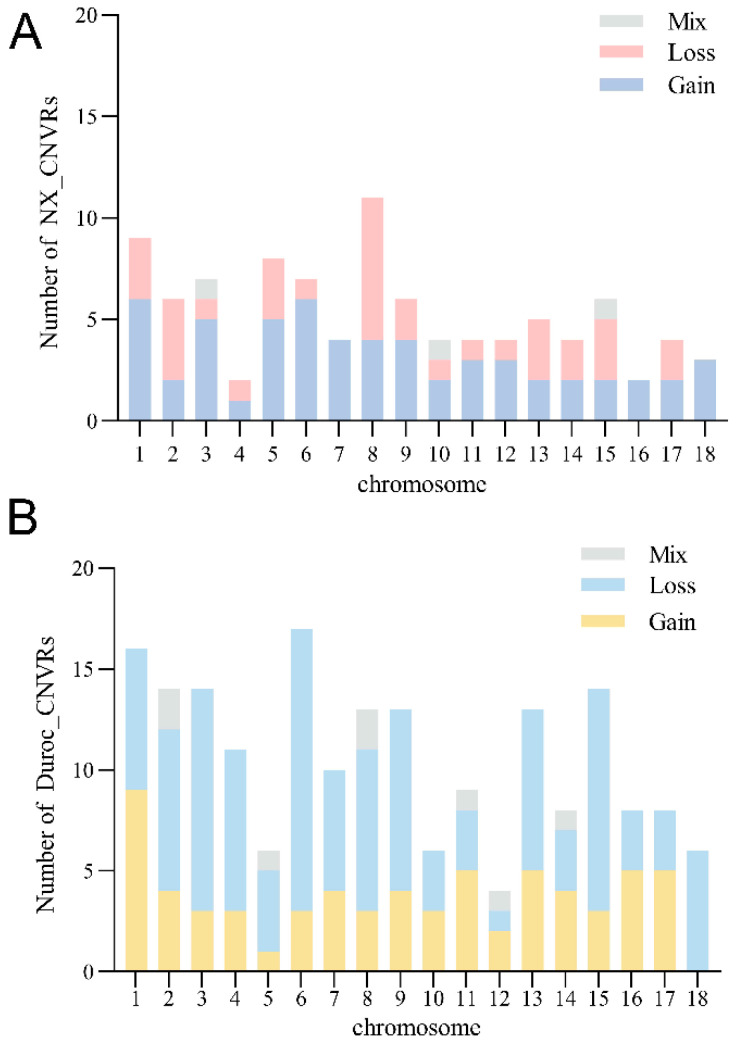
The composition and distribution of CNV types of CNVRs. The structure of the number distribution of CNV variation types of CNVRs on each chromosome of NX (**A**) and Duroc (**B**).

To determine whether the CNVs that we identified in NX differed from those in Duroc, the location and characteristics of all CNVRs are displayed in Figure 3A and Figure 4A, and the numbers of CNVRs in each autosome are presented in Figure 3B and Figure 4B. We identified 98 CNVRs, comprising 12.19 Mb in NX, and accounting for 0.49% of the whole genome (Figure 3A). The longest total length of CNVRs on SSC2 is 1,220,519 bp, and the shortest total length of CNVRs on SSC4 is 86,578 bp; furthermore, the coverage rate of CNVRs on chromosomes is only 0.1% (Table 2 and Figure 3A). These CNVRs were further divided based on their CN into gain (*n* = 60), loss (*n* = 35), and mix (*n* = 3), comprising 7.64 Mb, 4.08 Mb, and 0.48 Mb, respectively. A total of 192 CNVRs, comprising 40.18 Mb, were identified in Duroc, accounting for 1.61% of the genome (Table 2 and Figure 3B). The total length of CNVRs in SSC2 was the longest, which was consistent with the results of NX. The number of CNVRs in the gain, loss, and mix categories were 68, 116, and 8, respectively, which were different from the distribution characteristics of CNVRs in NX.

According to the definition of shared CNVRs, it was found that twenty-one CNVRs overlapped in both populations, while five CNVRs were the complete opposite type of variation in both populations, those being gain for NX and loss for Duroc. This result suggests that NX and Duroc are consistent as the same species, despite inconsistencies in domestication history and the intensity of selection. It also shows that the results of this study are robust.

### 2.3. Population Genetics and Positive Selection Analysis

**Figure 5:**
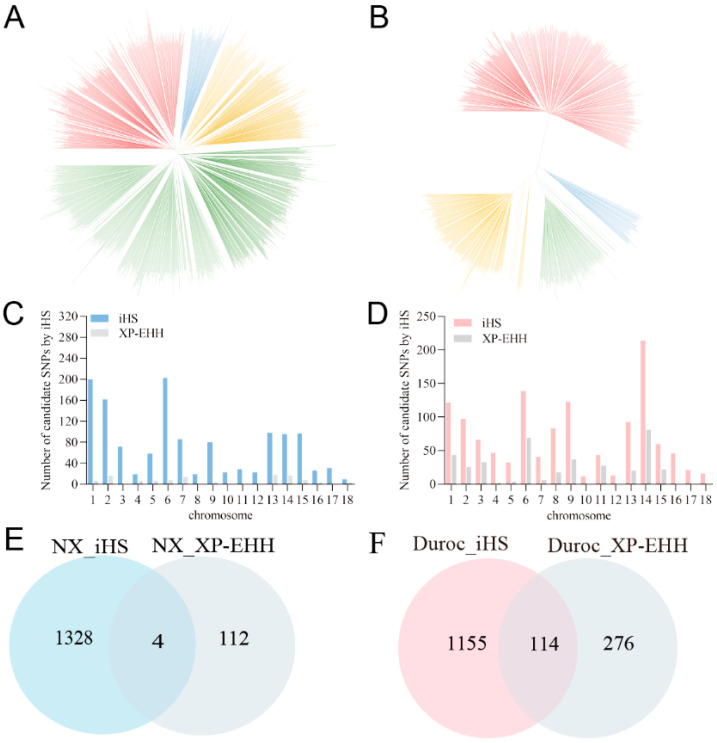
Comparison of genetic diversity and positive selection signal screening between NX and Duroc pigs. Phylogenetic tree of NX (**A**) and Duroc (**B**). The selection signals were calculated using IHS and xpehh methods, showing the distribution of candidate SNPs on chromosomes in Ningxiang (**C**) and Duroc pigs (**D**). In order to obtain a reliable selection signal, the SNPs selected by both methods are the final selection signals of NX (**E**) and Duroc (**F**).

Genetic diversity is important for population improvement and breed conservation in many ways, and it is influenced by domestication and selection. NX, as a native pig breed in southern China, has richer genetic diversity than exotic breeds. The phylogenetic tree showed that the NX population had more subgroups (Figure 5A), while Duroc was divided into two large branches (Figure 5B).

By comprehensively utilizing all of the genomic feature information of selection signals, a more comprehensive and accurate revelation of selection signals within the genome can be achieved. Selection signal methods based on the same theoretical basis can mutually verify and avoid the occurrence of false-positives in selection signals. Therefore, for better studying the similarities and differences between the two breeds, we used iHS and XP-EHH to conduct selective signal detection. The potential selection sites are unevenly distributed on the SSC1–SSC18 of the two breeds. By iHS, the most abundant selective signal was found on SSC12 in Duroc, whereas in NX it was SSC1, SSC2, and SSC6 that were more abundant. For the sake of accuracy in screening, we also used the XPEHH method to screen the data of the two populations (Figure 5C,D). From the dataset selected using two methods, there are 4 candidate-selected SNPs in the NX population, and 114 SNPs in the Duroc population (Figure 5E,F, Table S2). In the end, four selected regions were defined for NX, and 72 for Duroc.

### 2.4. CNV and Selection Regions’ Colocalization with Genes and Complex Trait QTLs

**Figure 6:**
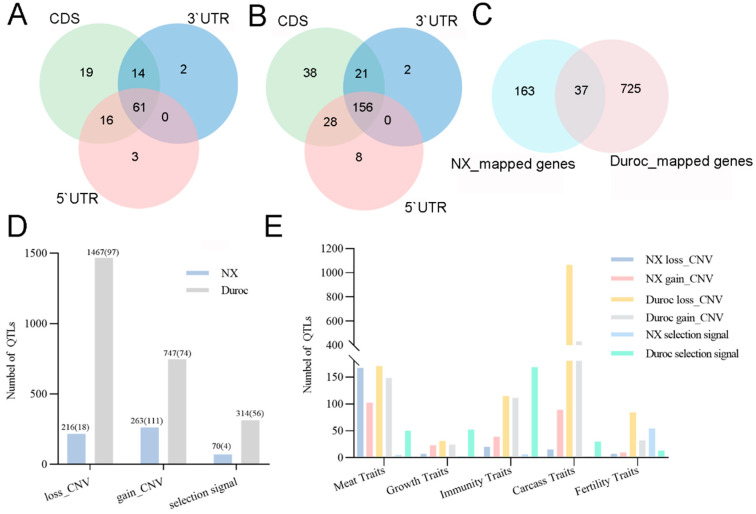
CNV and selection signal genome features. Venn diagram of 5’UTR, 3’UTR, and CDS (protein-coding region) of NX (**A**) and Duroc pigs (**B**). (**C**): Genes shared by Duroc and NX. (**D**): The number of QTLs mapped by CNVs and selection regions; the numbers in brackets represent the number of SNPs; and the numbers in front of brackets represent the number of QTLs. (**E**): The number of QTLs annotated on the main traits of different types of CNVs and selection regions in NX and Duroc (**E**).

The potential effects of CNV and the selection signal on the genome function were examined by conducting overlapping tests with distinct genome features, including genic sequence, regulatory variants, and complex trait QTLs in pigs. To investigate the impacts of CNVs and selection regions occurring in the genomic region, we annotated those CNVs and selection regions using genes (Table 3). CNVs, as long fragment variants, are much more likely to affect genes and their expression than single nucleotide variants. Different locations of occurrence have different effects on genes and their expression, and copy number variation rarely occurred in the 3`UTR (Figure 6A,B). In the NX population, 84 of 110 CDS-mapped CNVs were strong impact variants (84/168), and 103 promoter-mapped CNVs were likely to have a strong impact on gene function. This occurred in the case of Duroc, where 168 of the 243 CDS-mapped CNVs were strong impact variants, and 221 promoter-mapped CNVs were recognized as being in the strong impact class. In all, 87 (51.79%) CNVs of NX and 201 (61.65%) CNVs of Duroc covered 200 (63 CNVs annotated to 128 protein-coding genes) and 762 (163 CNVs annotated to 534 protein-coding genes) genes, respectively (Figure 6C). Furthermore, three and sixty-four (88.89%) selected regions of NX and Duroc, respectively, occurred in the CDS region. There were no strong effects of CDS-mapped CNVs in the NX population, whereas 32 strong effect CNVs were localized in the Duroc population. Interestingly, the CNVs localized to promoter regions all had strong effects. The selection regions of NX and Duroc were annotated to 37 and 319 genes, containing 24 and 238 protein-coding genes, respectively.

QTL and gene annotations were performed for population-specific and population-shared CNVs and selection regions, respectively, as defined. Both CNVs and selection regions of NX and Duroc were annotated to QTLs related to meat quality, growth, immunity, carcass, reproduction, and other traits. In all, 129 (76.79%) CNVs in NX pigs were annotated to 479 QTLs, which were mainly related to meat quality (269) and carcass traits (104). The meat quality traits were mainly muscle shear-related QTLs, including 33 gain CNV-mapped QTLs and 90 loss CNV-mapped QTLs. It was observed that most gain CNV-mapped QTLs of NX were predominantly located on SSC2 and SSC7, with these specific chromosomal fragments being primarily associated with meat quality and unsaturated fatty acid content (Tables S3 and S4). Duroc’s 171 (52.45%) CNVs annotated to 2214 QTLs, mainly associated with carcass (1499) and meat quality traits (318). There were 432 gain CNV-mapped QTLs and 1067 loss CNV-mapped QTLs for carcass traits. It is worth noting that 944 loss CNV-mapped QTLs were related to rib number, and there was a deletion of the SSC7:96457374-96806775 fragment (Figure 6D,E). Meat- and carcass-related QTLs obtained from the localization of CNVs in both populations were the most abundant. Further analysis showed that the distribution pattern of CNV types in the meat quality group was consistent in both populations, i.e., there were more deletion CNVs, while in the carcass group, the two populations had the opposite patterns of CNV-type distribution, with gain CNVs annotated with more carcass-related QTLs in NX, and deletion CNVs in Duroc (Figure 6E). This may be caused by the fact that Duroc experienced a greater intensity of artificial selection aimed at improving carcass performance.

There were seventy QTLs in the four selected regions of NX. These QTLs are mainly distributed on SSC13 (52), including the longest gestation length QTLs (Figure 6E, Table S3). Furthermore, 56 of the 72 selected regions in Duroc localized 314 QTLs, with the highest distribution of QTLs on chromosomes SSC3 and SSC14 (Figure 6D). Further comprehensive analysis of CNVs and the selected regions on the genome indicated that there were three common QTLs in Duroc. These are the intramuscular fat content QTL (147426), the days to 100 kg QTL (62329), and the oiliness QTL (164972). The former two lie within the interval gain CNV (SSC11:43791486-43890386), and the latter lies within the interval deletion CNV (SSC9:72211466-72462599).

### 2.5. Gene Annotation and Enrichment Analysis

**Figure 7:**
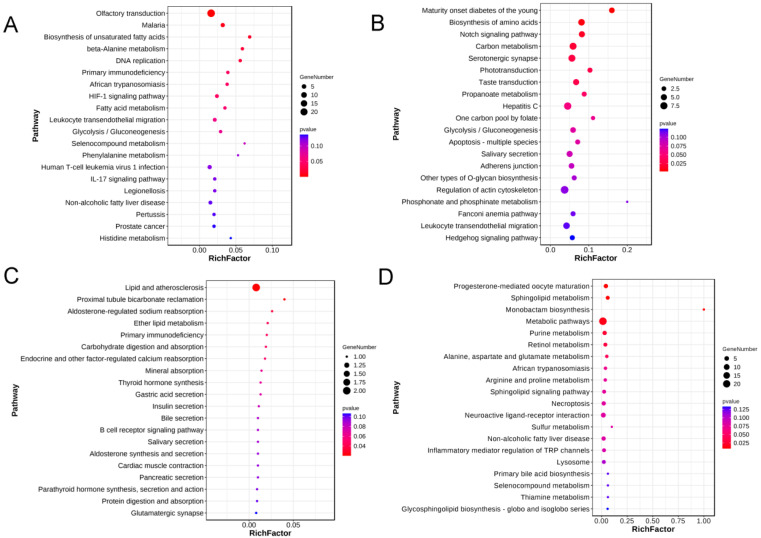
The top 20 KEGG pathways with enrichment of candidate genes in CNVs and selection regions in NX and Duroc. (**A**): CNV-mapped genes in NX. (**B**): CNV-mapped genes in Duroc. (**C**): Genes located in selection regions in NX. (**D**): Genes located in selection regions in Duroc.

Functional annotation and enrichment analyses were performed on CNV-mapped genes and selection region genes for NX and Duroc. The results of the KEGG enrichment analysis of CNV and selection regions in NX and Duroc are shown in the Figure below. The CNVs in Ningxiang pigs are mainly enriched in pathways such as immunity, fatty acid synthesis, and metabolism, including the biosynthesis of unsaturated fatty acids, fatty acid metabolism, and primary immunodeficiency. The enrichment analysis of CNVs in NX revealed that a total of 200 genes and 400 GO terms were significantly enriched, and that there are 128 genes that were enriched to 166 KEGG pathways (Figure 7A and Tables S5 and S6). The results showed that Duroc had 762 CNV-mapped genes, of which 534 were enriched to 258 KEGG pathways; furthermore, 486 were enriched to 5854 GO terms, of which 614 were significantly enriched. The CNV-mapped genes in Duroc pigs are mainly enriched in pathways related to sugar and carbon metabolism, amino acid synthesis, and taste transduction, such as carbon metabolism, biosynthesis of amino acid, glycolysis/gluconeogenesis, and taste transduction (Figure 7B and Tables S7 and S8).

For share CNVs, we classified them into the following two groups, according to the variant types: differential CNVs and consistent CNVs. We also analyzed the KEGG enrichment for both groups. Twenty-five genes in the differential CNVs were enriched for twenty-seven KTGG pathways, the main function of which is fatty acid anabolism, including HACD and GPER1 genes (Tables S9); furthermore, seventeen of the fifty genes in consistent CNVs were highly significantly enriched in the olfactory transduction pathway (Tables S10).

Furthermore, enrichment analysis of the genes located in selection regions in both populations revealed some interesting findings. In NX pigs, 24 genes out of 37 genes were enriched in 42 KEGG pathways, and 19 genes were enriched in 936 GO terms (Figure 7C and Tables S11 and S12). KEGG enrichment analysis of the selection regions of Ningxiang pigs also found that most genes were related to fat metabolism, immunity, and digestion and absorption, such as the following: lipid and anthero sclerosis, primary immunodeficiency, and carbohydrase digestion and absorption. In Duroc pigs, 522 GO terms were significantly enriched. Out of a total of 380 genes, 201 genes were enriched in 198 KEGG pathways, such as sphingolipid metabolism, monobactam biosynthesis, and purine metabolism (Figure 7D and Tables S13 and S14). The most significant enrichment factor was the monobactam biosynthesis signaling pathway, while a large number of genes were also enriched in signaling pathways related to physiological functions, such as the following: progesterone-mediated oocyte maturation, sphingolipid metabolism, and metabolic pathways.

## 3. Discussion

### 3.1. Differences in IMF Content and Growth Rate Between Duroc and NX Pig Breeds

Duroc, as an exemplary representative of American breeds, exhibits high IMF content and an exceptional growth rate. In contrast, NX, despite possessing similar levels of IMF content, displays a slower growth rate [2]. To investigate the similarities and differences between these two breeds, we based on CNV to analyze both populations with respect to their chromosomal positions, as well as their frequency. Combined with the screening of selection signals, the potential relationship between selection, variation, and phenotype was explored. Given the initiation of wild boar domestication in Asia and Europe around 10,000 years ago, various species have experienced adaptive changes in response to both environmental shifts and specific human needs [20]. The interplay between environmental pressures and intentional human selection has led to a diverse range of phenotypic variations within different breeds. This diversity is primarily attributed to contemporary conscientious selective breeding techniques. Duroc and NX, as representatives of early and recent selection, have intuitive manifestations in the detection of selection signals. Because of the model characteristics, iHS and XP-EHH are more sensitive to the recently selected signals [21]. Studies have shown that selection may promote the occurrence of genomic structural variation [22]. Our results also suggest that Duroc pigs, having an earlier and longer selection history, have richer copy number variations represented over a greater portion of the whole genome. This is consistent with the difference between Anhui local pigs and Western commercial pigs found by Xu et al. [23]. Therefore, it is crucial for researchers to focus on model selection and analyze CNVs when studying pig genetic variability, since these factors greatly impact pig phenotypes, and particularly their quantitative traits, as CNVs can disrupt gene dosage levels as well as expression patterns.

### 3.2. Correlation Between NX Pig CNV Mapping Gene and Fat Deposition Pathway

The study on the CNV-mapping gene of NX pigs found that multiple genes in NX pigs were enriched in the signaling pathway related to fat deposition, such as the following: *ACOX1*, *ACOX3*, *HACD*, and *VLCAD*. ACOX1 and ACOX3 (straight-chain acyl-CoA oxidase 1) are key enzymes in the oxidation of fat. Fat oxidation mainly includes the following three processes: oxidation of acetyl-CoA, oxidation of pyruvate, and oxidative phosphorylation, while ACOX1 is the rate-limiting enzyme in the oxidation of acetyl-CoA. Some studies have shown that the variation of the ACOX1 gene can significantly affect the meat quality traits of pork [24]. Furthermore, it has been found that the *ACOX1* is significantly correlated with QTLs, such as daily weight gain, backfat thickness, and fatty acid composition in pigs [25]. HACD (3-hydroxyacyl-CoA dehydratase) is a crucial enzyme that catalyzes the synthesis of long-chain to ultra-long-chain fatty acids, with its encoded protein primarily found in the endoplasmic reticulum of cells [26]. Knocking out the HACD1 gene in mice leads to a pronounced myopathic phenotype, and *HACD1* KO mice exhibit marked reductions in body size, weight, and skeletal muscle mass [27]. Very-long-chain (3R)-3-hydroxyacyl-CoA dehydratase (*VLCAD*) is a catalytic enzyme that catalyzes the oxidation of very-long-chain fatty acids. It is present in various tissues of the animal body. When mice lack CLCAD, they exhibit symptoms of fatty liver, and the expression level of PPAR-γ in the body significantly increases, indicating that fat begins to deposit in the mouse body, and the breakdown of very-long-chain fatty acids is hindered [28]. It has been suggested that the fat content and intramuscular fat content of NX pigs were correlated with CNV.

### 3.3. Duroc Pig CNV Analysis Reveals Enrichment of Fatty Acid Synthesis and Taste Transmission-Related Genes and Their Effects on Meat Quality

Through the enrichment analysis of CNV in Duroc pigs, CNV-mapping genes were found to be involved in pathways related to fatty acid synthesis and taste conduction. The metabolite 3-hydroxyisobutyric acid (3-HIB), a valine (branched-chain amino acid), promotes FA uptake. Additionally, HIBCH (3-hydroxyisobutyryl-CoA hydrolase) plays a role in the downstream catabolic pathways of leucine and isoleucine by removing the CoA group from 3-hydroxypropyl CoA, forming 3-hydroxypropionic acid, a precursor of acetyl CoA. Studies indicate that *HIBCH* can inhibit oxidative phosphorylation, thereby contributing to fat deposition [29]. ECHS1 (Short-chain enoyl-CoA hydratase) is a key enzyme in the mitochondrial matrix’s fatty acid β-oxidation pathway, catalyzing the hydration reaction of α, β-unsaturated thioester substrates, with crotonyl-CoA being the most reactive substrate. Peng et al. discovered that overexpression of the ECHS1 gene enhances fatty acid oxidation, subsequently decreasing fat deposition in broiler chickens [30]. *HIBCH* and *ECHS1* are both enriched in the pathway of carbon metabolism. Through an analysis of the shared CNV genes in NX pigs and Duroc pigs, it was found that the genes of the shared CNVs were mainly enriched in pathways such as fatty acid elongation and the biosynthesis of unsaturated fatty acids. Therefore, it can be inferred that there are CNVs related to fat deposition in both NX and Duroc pigs, which may be one of the reasons why both breeds have good meat quality.

### 3.4. CNV Variation of Duroc Pig Taste-Related Genes and Its Impact on Meat Quality and Feed Preference

Pigs possess a more acute olfactory sense than numerous species, including humans, and this directly influences the feeding behaviors and reproductive patterns of mammals [31]. *TAS1R3* (Taste 1 Receptor Member 3) is a gene that encodes a protein responsible for taste receptor perception. It can associate with *TAS1R2* to establish a receptor for sweet taste responses, thus identifying the umami and sweet flavors in food. Variations in TAS1R3 across species contribute to differences in animals’ food selectivity and specificity [32]. The protein encoded by *TRPM5* (Transient Receptor Potential Cation Channel Subfamily M Member 5) is a key player in taste transmission, being part of the transient receptor potential protein family. Anthony Sclafani and colleagues’ research revealed that, relative to mice with their *TRPM5* knocked out, wild-type mice exhibit a greater tolerance for fats, whereas *TRPM5* KO mice demonstrate reduced sensitivity to low-concentration oily foods. Thus, it is inferred that the TRPM5 gene influences the animals’ dietary preferences for oily foods [33]. The protein subunit encoded by the GNB3 (guanine nucleotide-binding protein beta-3) gene is regarded as a crucial regulatory factor for signal transduction receptors and effectors, with reports indicating a significant association with obesity [34]. These genes exhibit copy number gain variations in Duroc pigs, leading to the inference that, compared to NX pigs, Duroc pigs possess more sensitive taste buds, which may contribute to their lower tolerance for roughage.

### 3.5. Differential Analysis of Selection Signals in Digestion- and Absorption-Related Pathways Between NX Pigs and Duroc Pigs

The selected regions of NX are predominantly located on SSC6 and SSC14, and there are a total of 37 annotated genes in NX pigs. In this study, there is no overlap observed between the selected region and CNVR in NX. It may be that there is a problem of sequencing depth, or it may be that the selection time is relatively close and has not yet been linked to genomic structural variation. The NX pigs’ selective signals are predominantly in pathways linked to digestion and absorption, including carbohydrate digestion and absorption, endocrine-regulated calcium reabsorption, gastric acid secretion, and bile secretion. Meanwhile, Duroc’s signals are concentrated in pathways concerning amino acid metabolism and biosynthesis, such as alanine, aspartate, and glutamate metabolism, and monobactam biosynthesis. This suggests that, under the combined selective pressures of human-induced and environmental factors, individuals with enhanced digestion and absorption possess a distinct advantage.

The use of 50 k chips to detect large structural variations, especially CNVs, is not sufficient. At the same time, the 50 k chips were not designed for the Chinese native pig population. These two reasons will lead to certain limitations in this study.

## 4. Methods and Materials

### 4.1. Animals

The NXs in this study came from Hunan Chuwei Xiang Agriculture and Animal Husbandry Co., Ltd. (Ningxiang, China) and the NX Breed Reserve, with a total of 1805 pigs. The Duroc pigs (Duroc) data were obtained from Gao et al. [35]. All animal tests comply with the regulations of The Animal Ethics Association of Hunan Agricultural University.

### 4.2. Genotyping, Genotype Imputation, and Quality Control

The total DNA of NXs were extracted from ear tissues or fresh semen using the traditional phenol/chloroform method. Concentration and purity of genomic DNA were assessed using a NanoDrop™ 2000 (Thermo Fisher Scientific, Waltham, MA, USA). High-quality DNA samples were genotyped using the GeneSeek Genomic Profiling (GGP) version 2 porcine 50 k SNP chips, containing 50,697 SNP loci (Neogen, Lincoln, NE, USA). To improve the timeliness of the research results, the physical positions of SNPs on the chip were updated to Sscrofa 11.1 for subsequent analysis. GenomeStudio (Illumina, version 2.0.5) was used for genotype calling and SNP clustering. Furthermore, strict quality control was used for SNP filtering to increase the accuracy of CNV detection. SNPs and individuals with a call rate of <90% were eliminated. SNPs with unknown positions were directly deleted, and if the positions of multiple SNPs were duplicated, then 1 of them was retained. Ultimately, 48,858 SNPs were obtained for subsequent analysis. The genotyping module of GenomeStudio soft (Illumina, Inc., San Diego, CA, USA) was used to determine the genotype signal intensity of the individuals, including log R ratio (LRR) and B-allele frequency (BAF).

Due to the small size of the NX population, the sample sources were scattered. In order to ensure that the CNVs were representative of the population, the GBC was first estimated for the individual NX samples, with GBC > 0.94 for purebred NX, while non-NX individuals were excluded [3]. The individuals with a GBC value of less than 0.94 were excluded, resulting in 1773 remaining individuals. CNV detection was performed on these 1773 individuals.

### 4.3. Genome-Wide Detection of CNVs and CNVRs

The population frequency (PFB) files were created based on the individuals from the NXs’ core breeding farm, and a total of 384 NX individuals were adopted. Duroc’s PFB file is directly generated from an SNP dataset of 1137 individuals. To salvage the sample affected by a genomic wave, a porcine GCmodel file was created by calculating the GC content of the 1 Mb region surrounding each SNP, and the -gcmodel option in PennCNV was used for adjustment. PennCNV cal_gc_snp.pl and wig2gc5base are used to obtain the GCmodel file. The wig file is derived from UCSC (https://hgdownload.soe.ucsc.edu/goldenPath/susScr11/ accessed on 12 January 2024), and genomic waves were adjusted for the GC content in the 500 kb genomic region around each SNP on both sides. Subsequently, each sample was subjected to quality control before analysis to reduce the possibility of false-positive CNVs. In summary, we included samples with LRR 0.3, BAF drift 0.01, and LRR 0.05 for the GC wave factor. Effective CNVs were classified as having at least two individuals, and three or more consecutive SNPs. Furthermore, the CNVs were divided into four categories based on the copy number, which are as follows: CN0, CN1, CN3, and CN4. The numbers represent the number of copies.

The copy number variation regions (CNVRs) are widely defined as the same genomic region of copy number variations that exist between different individuals. In this study, CNVRs were generated via the call_cnvr () function in the R package HandyCNV (version 1.1.7), as the union of sets of CNVs that overlap by at least one base pair. The detection rate of CNVs in a population (R-CNV) is defined as the number of detected CNVs in a population, divided by the total number of individuals in the population.

### 4.4. Genetic Diversity and Genetic Structure

FastME provides distance algorithms to infer phylogenies. FastME improves over the Neighbor-Joining method by performing topological moves using fast, sophisticated algorithms [36]. Interindividual distances (VCF2Dis, https://github.com/hewm2008/VCF2Dis, accessed on 18 January 2024) were first calculated to obtain a distance matrix. The distance matrix was then used as input for the ATGC (http://www.atgc-montpellier.fr/fastme/, accessed on 20 January 2024) web tool for phylogenetic inference. Finally, the phylogenetic tree was visualized using iTOL (https://itol.embl.de/upload.cgi, accessed on 20 January 2024).

### 4.5. Identification of Genomic Selected Regions

After quality control (plink1.9 –maf 0.01 –hwe 0.00000001 –mind 0.1 –geno 0.1), 22,620 and 22,504 SNPs were finally used for the subsequent analysis of NX and Duroc, respectively. In order to provide a reliable level of results, this study used the R package rehh v 3.2.2 [37] to calculate iHS and XP-EHH as two indicators for signal analysis. The Integrated Haplotype Score (iHS) is defined as the area under the EHH curve, which is defined by the EHH values and associated chromosomal positions. Cross Population EHH (XP-EHH) is based on the principle of genetic linkage imbalance. After artificial selection and improvement, the population will undergo a large range of chromosomal recombination. Due to the existence of linkage, neutral sites near the mutated gene will also be transmitted generation by generation, resulting in the formation of a longer range of haplotype homozygosity on the chromosome. The first tests detect partial selective sweeps, whereas XP-EHH detects selected alleles that have risen to near fixation in one but not all populations. Using NX pigs as the experimental group and Duroc pigs as the reference group, we calculated the XP-EHH values. Afterward, a candidate selected regions, extending 200 kb upstream and downstream of the local SNP, is defined.

### 4.6. Validation of the Accuracy of CNV Call

To verify the CNVs identified by PennCNV v1.0.5, we used the QuantiSNP v.2.3 software [38] to analyze the NX’s data. The QuantiSNP algorithm uses an Objective Bayes Hidden-Markov model to improve the accuracy of the CNVs’ identification and mapping, and uses a fixed rate of heterozygosity for every SNP. This CNV calling software was run under default parameters.

### 4.7. Annotations for CNVs and Selected Regions

To further analyze the possible phenotypic effects of CNV and selection signals in NX and Duroc pigs, we defined population-specific CNVs and selection signals as events detected only in one population. If they were detected in both populations, they were defined as shared CNVs and selection signals. In this study, the BioMart tool (https://useast.ensembl.org/biomart/martview/, accessed on 24 January 2024) of the online website Ensembl was utilized to identify candidate genes within CNVRs and the selected regions for subsequent enrichment analysis. Among them, Sscrofa11.1 was selected as a reference, and the annotation of candidate genes was based on the standard of “the physical position of genes on chromosomes overlaps with the target region” [39]. The online websites DAVID (https://david.ncifcrf.gov/summary.jsp, accessed on 24 January 2024) and gprofile (https://biit.cs.ut.ee/gprofiler/gost, accessed on 25 January 2024) were used to enrich genes into known functional pathways (terms), respectively; they were tested for significance (*p* < 0.05) and bonferroni multiple tests were used to improve the confidence of the results. Two databases, Gene Ontology (GO; https://www.geneontology.org/, accessed on 28 January 2024) and the Kyoto Encyclopedia of Genes and Genomes (KEGG; http://www.genome.jp/kegg/, accessed on 28 January 2024), were included.

To obtain the QTL distributions of the target regions, bed format annotation files were obtained from Pig QTLdb (Pig Quantitative Trait Locus (QTL) Database, (https://www.animalgenome.org/cgi-bin/QTLdb/SS/index, accessed on 28 January 2024)). Then, QTLs of CNVRs and selected regions were extracted by writing R code independently.

We classified those gene-overlapping CNVs and selection signals into seven categories, based on their location within a gene, including introns, exons, a cis-window (1 kb upstream and downstream of the transcription start site), promoter, 5′UTR, 3′UTR, and CDS (protein-coding region). Effective localization of genomic regions is based on a threshold of 20% coverage of the target genomic region by CNVs. CNV-mapped regions and selection signals were defined as having a strong (St) impact when overlapped with CDS accounts for greater than 50%.

## 5. Conclusions

Chinese native pigs and Western commercial pigs have formed their germplasm characteristics due to social and economic development, and geographical environment changes. In this study, NX and Duroc pigs were used as the research objects. Based on the genome-wide SNP genotyped data, the copy number variation atlas of the two breeds was constructed, and the candidate selection regions were screened. It was found that there were significant differences in the region and frequency of the copy number variation between NX and Duroc. A total of 98 CNVRs were identified in NX pigs, while 192 CNVRs were identified in Duroc pigs, which exhibited higher genome coverage compared to NX. Additionally, 21 CNVRs overlaps were found between the two breeds, with a majority of these overlaps occurring in pathways related to fat metabolism, based on enrichment analysis. Selection signal analysis revealed that NX pigs displayed more subpopulations than Duroc. QTL annotation results indicated that gain CNV in NX pigs and deletion CNV in Duroc pigs both annotated carcass-related QTL, potentially linked to the relatively isolated geographical environment of NX pigs, and the stronger manual selection experienced by Duroc pigs. We explored this change to provide new information for the domestication history of NX, and the analysis of the genetic basis of germplasm characteristics, which is conducive to the protection of germplasm resources of Chinese native pigs, and accelerates the progress of genetic improvement.

## Figures and Tables

**Table 1:** Sample quality control and effective CNVs.

Group	N Unique Pigs		nCNV (Unique Pigs)		
	Before QC	After QC	Total	Loss	Gain
NX	1773	1194	168 (834)	57	111
Duroc	1137	1011	326 (828)	203	123

The numbers in parentheses represent the number of individuals with an effective copy number variation.

**Table 2:** Statistics of CNVRs identified in NX and Duroc pigs.

	Ningxiang Pigs			Duroc Pigs		
	Gain	Loss	Mix	Gain	Loss	Mix
N	60	35	3	68	116	8
Min length (bp)	5285	17,781	114,380	23,656	23,897	259,803
Max length (bp)	482,505	380,658	223,221	1,300,628	774,622	1,844,046
Average length (bp)	127,293	116,527	158,535	148,770	202,103	825,891
Total length (bp)	7,637,595	4,078,451	475,606	10,116,388	23,443,980	6,607,128

N, number of CNVRs; Gain, increased copy number; Loss, loss of copy number; Mix, CNVR has both the copy number increase variation and copy number deletion variation in this population.

**Table 3:** Distribution of CNVs and selected regions on genomic structure.

	BREED	3utr	5utr	CDS	CDS_ST	Cis_Window	Exons	Introns	Promoter	Promoter_ST
selection regions	NX	3	2	3	0	4	4	4	4	4
	Duroc	61	57	64	32	62	70	67	67	67
CNV regions	NX	77	0	110	84	92	133	115	103	103
	Duroc	179	192	243	168	223	275	262	221	221

Figures indicate the number of CNVs/selected regions localized to the structural domain. The “ST” character in the column name indicates that the CNVs/selected regions in the column have strong effects.

## Data Availability

All data generated or analyzed during this study are included in this published article.

## Supplementary Materials

The supporting information can be downloaded at: https://www.mdpi.com/article/10.3390/ijms252111716/s1.
